# Case Report: Camrelizumab combined with gemcitabine and oxaliplatin in the treatment of advanced intrahepatic cholangiocarcinoma: a case report and literature review

**DOI:** 10.3389/fimmu.2023.1230261

**Published:** 2023-08-21

**Authors:** Zhongyan Zhang, Xin Wang, Hehe Li, Huimin Sun, Jianhong Chen, Hongfeng Lin

**Affiliations:** ^1^ Department of Hepatobiliary Surgery, Weifang People’s Hospital, Weifang, China; ^2^ Department of Geriatrics, Weifang People’s Hospital, Weifang, China; ^3^ Department of Pathology, Weifang People’s Hospital, Weifang, China

**Keywords:** advanced intrahepatic cholangiocarcinoma, immunotherapy, chemotherapy, conversion therapy, surgical resection

## Abstract

Intrahepatic cholangiocarcinoma (ICC) is one of the most common invasive malignant tumors, with a 5-year survival rate of less than 5%. Currently, radical surgical resection is the preferred treatment for ICC. However, most patients are only diagnosed at an advanced stage and are therefore not eligible for surgery. Herein, we present a case of advanced ICC in which radical surgery was not possible due to tumor invasion of the second porta hepatis and right hepatic artery. Six treatment cycles with a gemcitabine and oxaliplatin (GEMOX) regimen combined with camrelizumab immunotherapy achieved a partial response and successful tumor conversion, as tumor invasion of the second porta hepatis and right hepatic artery was no longer evident. The patient subsequently underwent successful radical surgical resection, including hepatectomy, caudate lobe resection, and cholecystectomy combined with lymph node dissection. Cases of patients with advanced ICC undergoing surgical resection after combined immunotherapy and chemotherapy are rare. The GEMOX regimen combined with camrelizumab demonstrated favorable antitumor efficacy and safety, suggesting that it might be a potential feasible and safe conversion therapy strategy for patients with advanced ICC.

## Introduction

Intrahepatic cholangiocarcinoma (ICC) is a malignant tumor originating from the secondary bile ducts and their branches in the liver. It is the second most common malignant tumor of the liver, accounting for 5–20% of all malignant liver tumors. The 5-year survival rate is less than 5% (1). Surgical resection is the preferred treatment for ICC; however, because of its insidious onset, most patients have lymph node or distant metastases at the time of diagnosis and cannot undergo surgical resection (2). Moreover, the recurrence rate after surgical resection is very high and the prognosis is poor. For patients with advanced, inoperable ICC, the National Comprehensive Cancer Network (NCCN) recommends gemcitabine-based chemotherapy combined with platinum-based drugs as first-line treatment; however, the overall efficacy is poor and radical surgical resection remains essential to prolong survival. Increasing R0 resection rates is therefore vital for improving the prognosis of patients with ICC. Conversion therapy aims to convert unresectable tumors into resectable tumors through systemic therapies, such as chemotherapy. Tumors are initially treated with chemotherapy to reduce their size, thus enabling radical surgical resection (3, 4). ICC is an aggressive malignant tumor with a complex tumor microenvironment and high metastatic potential, and many conventional treatment modalities, including chemotherapy and radiotherapy, exhibit limited efficacy for treatment of ICC. Programmed cell death protein 1(PD-1) and programmed cell death 1 ligand 1(PD-L1) are immune checkpoint proteins that downregulate immune responses, and immune checkpoint inhibitors (ICIs), especially PD-1/PD-L1 inhibitors, have shown promising outcomes for treating various cancers (5). PD-L1 expression has been detected in approximately 30% of ICC cases, primarily in stromal cells (6–8). Herein, we describe a case of advanced stage ICC treated with camrelizumab combined with a gemcitabine and oxaliplatin (GEMOX) regimen, followed by curative resection. We also review related literature to suggest that it might be a potentially feasible and safe conversion therapy strategy for patients with advanced ICC.

## Case description

**Figure 1:**
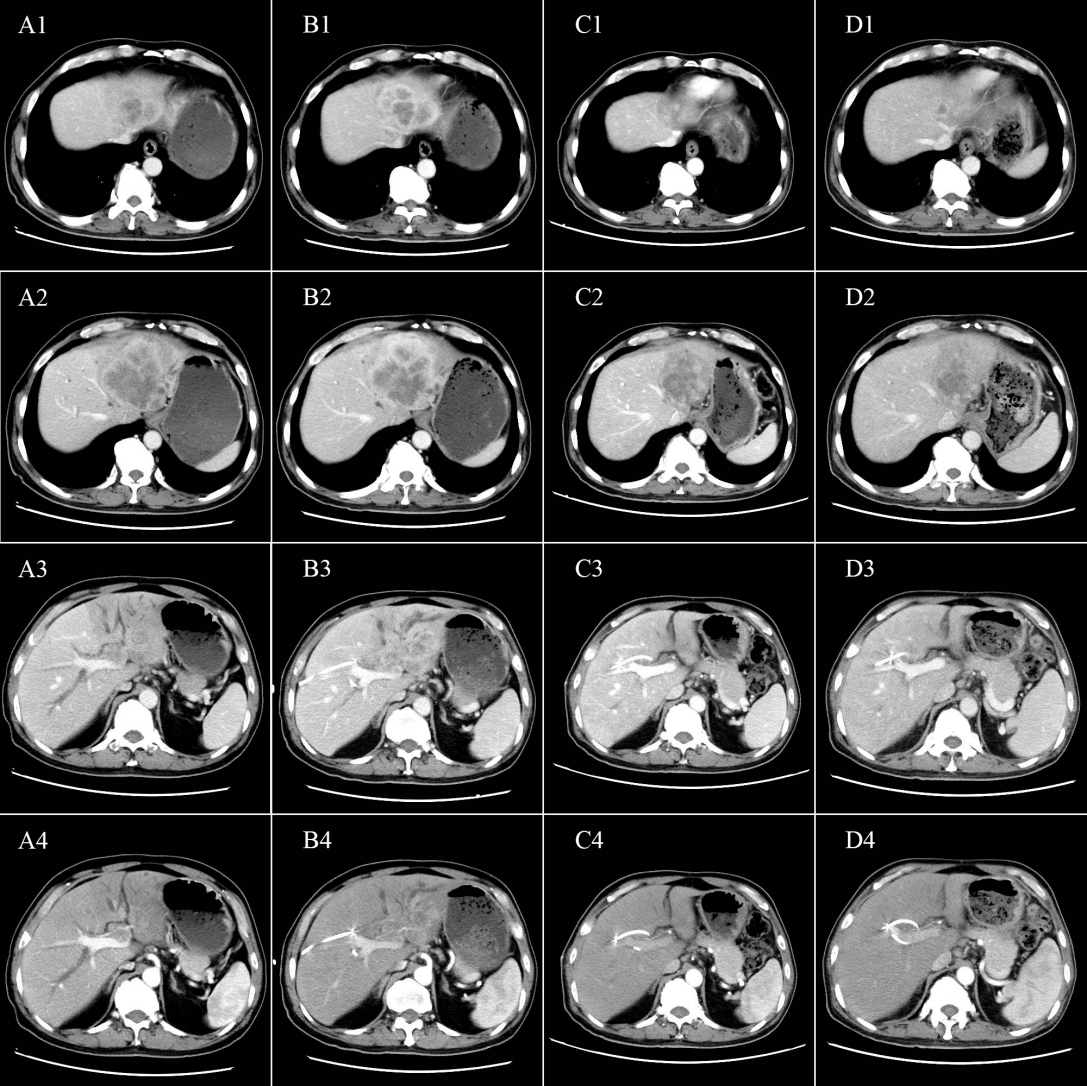
Tumor characteristics before and after conversion therapy. Relationship between the tumor and **(A1, B1, C1, D1)** the second porta hepatis; **(A2, B2, C2, D2)** the middle hepatic vein; **(A3, B3, C3, D3)** the first porta hepatis; and **(A4, B4, C4, D4)** the right hepatic artery. **(A1-A4)** Abdominal CT before conversion therapy shows tumor invasion of the middle hepatic vein, left branch of the portal vein, and the right hepatic artery. **(B1-B4)** After two cycles of conversion therapy, there are no significant changes in the tumor. **(C1-C4)** After four cycles of conversion therapy, the tumor is significantly smaller. **(D1-D4)** After six cycles of conversion therapy, the tumor is further reduced.

**Figure 2:**
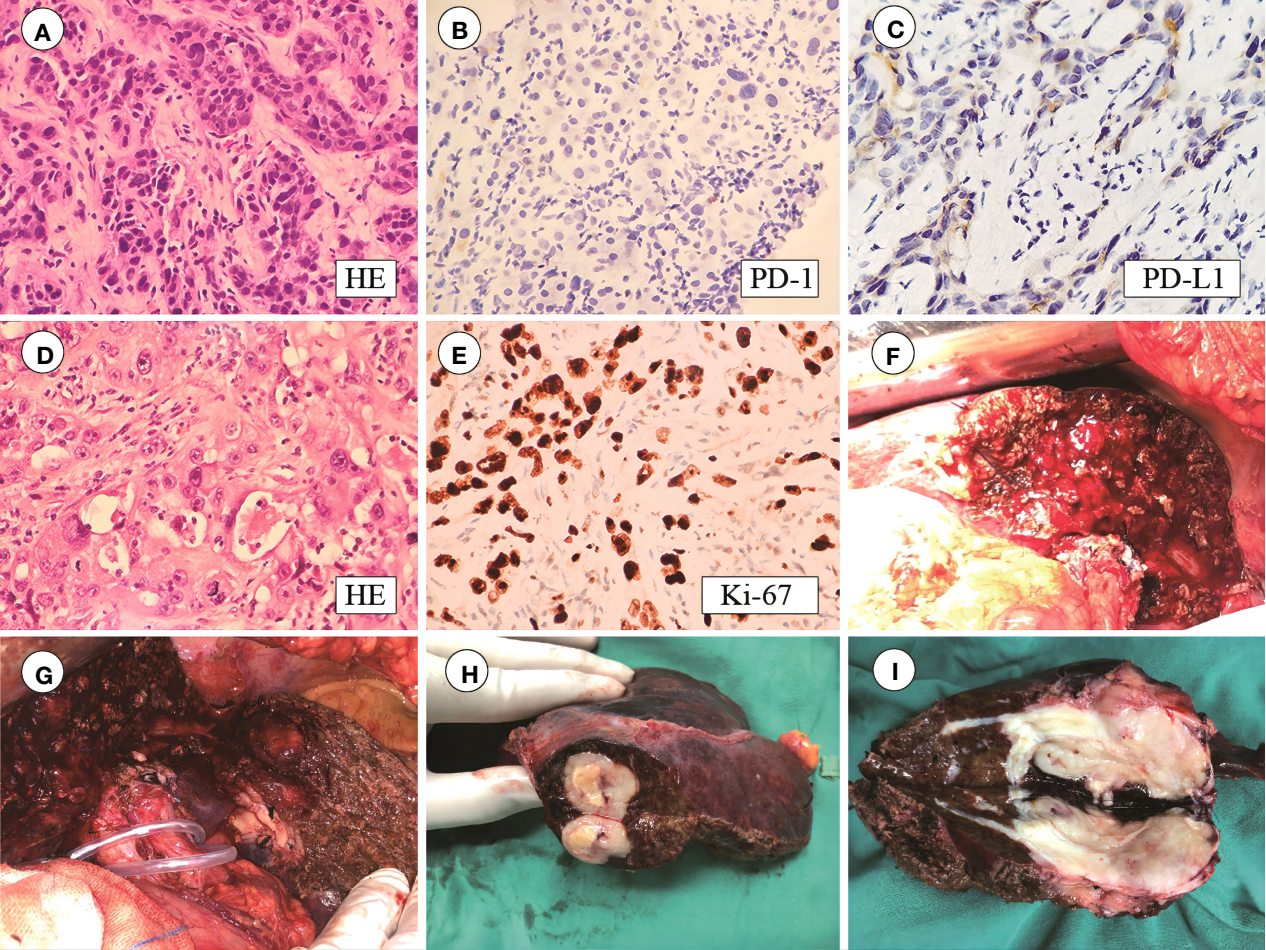
**(A)** HE staining of puncture tissue; **(B)** PD-1 staining is negative; **(C)** PD-L1 staining is positive in 30% of tumor cells and 5% of stromal cells; **(D)** Postoperative HE staining; **(E)** Ki-67 index 80%; **(F–I)** Tumor size 6.5 × 4 × 4 cm with capsular invasion.

In May 2020, a 64-year-old male was admitted to the hospital for dark brown urine persisting for more than 10 days. Physical examination revealed whole-body skin, mucous membrane, and scleral jaundice; upper abdominal tenderness; no rebound tenderness; no palpable abdominal mass; and no obvious enlargement of the subclavicular lymph nodes. On May 21, 2020, serum tests showed significantly elevated carbohydrate antigen 125 (CA125) (1276.30 U/mL, normal range 0–30.2 U/mL), significantly elevated carbohydrate antigen 19-9 (CA19-9) (677.45 U/mL, normal range 0–37 U/mL), elevated alanine aminotransferase (238 U/L, normal range 5–40 U/L), elevated aspartate aminotransferase (105 U/L, normal range 8–42 U/L), elevated total bilirubin (131.7 µmol/L, normal range 7.4–24.1 µmol/L), elevated direct bilirubin (95.3 µmol/L, normal range 0–6.8 µmol/L), and elevated indirect bilirubin (36.4 µmol/L, normal range 0–20 µmol/L) levels. A-fetoprotein (AFP) and carcino-embryonic antigen (CEA) levels were within normal ranges. Chest and abdominal enhanced computed tomography (CT) performed on May 21, 2020 (**Figures 1A1–A4**) revealed enlargement of the left lobe of the liver, internal and surrounding patchy low-density areas, blurred borders, uneven contrast enhancement, dilated intrahepatic bile duct, and enlarged lymph nodes in the porta hepatis and retroperitoneum. On May 22, 2020, percutaneous transhepatic cholangial drainage and ultrasound-guided liver tumor biopsy were performed. Pathological examination (**Figures 2A–C**) revealed bile duct cell carcinoma (moderately to poorly differentiated). Immunohistochemistry showed that the tumor tissues were positive for CK wide, CK19, CK7, MLH1, MSH2, MSH6, and PMS2, but negative for PAX-8, Syn, P40, HepPar-1, Glypican-3, TTF-1, CDX2, CK20, PSA, and PD-1. PD-L1 expression was 30% in tumor cells and 5% in stromal cells. ICC with invasion of the middle hepatic vein, left branch of the portal vein, and right hepatic artery, and biliary tract dilation (AJCC cT4N1M0 stage IIIb) was diagnosed.

**Figure 3:**
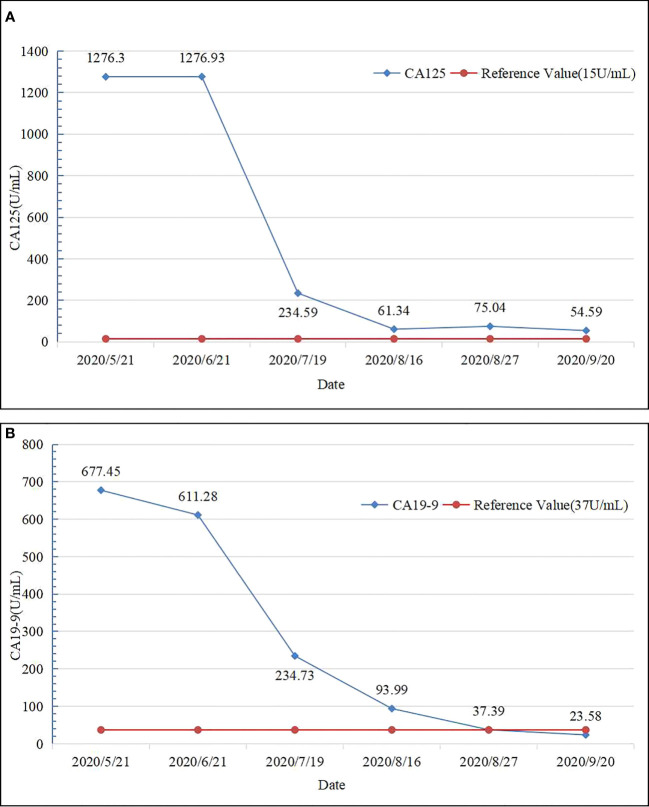
Tumor marker expression levels before and after chemotherapy. **(A)** CA125 levels before and after chemotherapy. **(B)** CA19-9 levels before and after chemotherapy.

On May 25, 2020, treatment with camrelizumab (200 mg, intravenous drip (ivd), D1/2W) plus gemcitabine (800 mg/m^2^, ivd, D1/2W) and oxaliplatin (85 mg/m^2^, ivd, D2/2W) was initiated. Treatment-related adverse events included decreased appetite, nausea, and peripheral sensory neuropathy, with no intolerable grade 3–4 treatment-related adverse events. All immune and chemotherapy-related adverse events were well-controlled and resolved after treatment. After two cycles of conversion therapy, there are no significant changes in the tumor (**Figures 1B1-B4**). But after four cycles of conversion therapy, the tumor is significantly smaller than before (**Figures 1C1-C4**). After six treatment cycles, abdominal CT showed that the tumor had reduced and no longer invaded the first porta hepatis, second porta hepatis, middle hepatic vein, or right hepatic artery (**Figures 1D1–D4**). Tumor marker expression gradually decreased (**Figure 3**). Treatment efficacy was evaluated as a partial response.

On August 17, 2020, the patient completed the last cycle of conversion therapy, routine blood, coagulation, and renal function test results showed no significant abnormalities. Alanine aminotransferase (49 U/L; normal range 5–40 U/L), aspartate aminotransferase (50 U/L; normal range 8–42 U/L), total bilirubin (16.5 µmol/L, normal range 7.4–24.1 µmol/L), direct bilirubin (9 µmol/L, normal range 0–6.8 µmol/L), and indirect bilirubin (7.5 µmol/L, normal range 0–20 µmol/L) levels were elevated.

On August 19, 2020, the patient underwent left hepatectomy, caudate lobectomy, cholecystectomy, and abdominal lymph node dissection. The surgery was uneventful, with an operative time of 270 min and an estimated blood loss of 200 mL. Postoperative pathological examination (**Figures 2D, E**) revealed moderate-to-poorly differentiated cholangiocarcinoma with multiple foci of calcification, extensive fibrosis, and collagen degeneration in the stroma, locally infiltrated by a large number of lymphocytes and plasma cells. Immunohistochemistry revealed that the tumor tissues were positive for CK7 and CK19, but negative for HepPar-1, Glypican-3, and AFP. The Ki-67 index was 80%.

The tumor had a volume of 6.5 × 4 × 4 cm and exhibited capsular invasion, but no obvious cancer emboli were observed in the vessels (**Figures 2F–I**). The surgical margins are clear. Spotty necrosis and cholestasis were observed in the surrounding small liver lobes. Lymphocytic infiltration was observed in the bile duct. No metastasis was found in Group 8 lymph nodes (0/5), and one lymph node exhibited calcification. Hepatic pedicle lymph nodes contained small amounts of liver tissue and cancer components. The tail lobe of the liver exhibited liver cell degeneration, cholestasis, and lymphocytic infiltration in the bile duct area, with no specific changes at the cut edge. The gallbladder exhibited acute and chronic cholecystitis and adenomyomatosis.

The patient was discharged 11 days after surgery after an unremarkable postoperative course. Following surgery, oral capecitabine (1250 mg/m^2^ daily from day 1 to day 14; then every 3 weeks) was started and continued until disease progression. Follow-up was carried out at 2-month intervals during the first year, and involved physical evaluation, blood cell count, blood biochemistry, tumor marker evaluation, and enhanced CT. The patient experienced tumor recurrence in October 2021 and did not receive further treatment at the local hospital. Unfortunately, the patient passed away in December 2021. The postoperative survival time was 16 months.

## Discussion

ICC is a common malignant tumor, and the second most common intrahepatic malignancy, accounting for 20% of all liver malignancies and 3% of all gastrointestinal malignancies (9). The NCCN guidelines classify ICC into resectable, unresectable, and metastatic disease, based on therapeutic considerations (10). Surgical resection with negative (R0) microscopic margins is the optimal therapy for ICC. However, less than 20–30% of patients are candidates for resection at the time of diagnosis because of locally progressed or metastatic disease (11, 12). Furthermore, the prognosis remains poor, with 5-year survival rates ranging from 20 to 35% after resection (13). Owing to the non-specific manifestations of early disease, most patients with ICC present at advanced stages when radical surgical resection is no longer feasible. By the time patients develop abdominal pain, jaundice, or other symptoms, only approximately 30% remain eligible for radical surgical resection (14). For patients with unresectable advanced ICC, the 5-year survival rate is less than 10% using the standard treatment. Therefore, improving the surgical resection rate is urgently required to improve the outcome of patients with ICC. The advancement of systemic therapy has increased number of ICC cases with successful conversion to resectable tumors. According to the ABC-02 trial (ClinicalTrials.gov identifier NCT01926236) published in 2010, the most effective first-line treatment for BTC is gemcitabine and cisplatin doublet chemotherapy (9, 15). However, new triplet regimens and immunotherapy may improve efficacy. In recent years, ICIs have been successfully used to treat hepatocellular carcinoma, and multiple studies have evaluated the efficacy of ICIs as monotherapy for biliary tract cancer (BTC) (16, 17). NCCN guidelines recommend PD-1 inhibition for ICC with mismatch repair deficiency (dMMR) and elevated microsatellite instability (MSI-H), and PD-L1 expression is a predictive biomarker for the response to ICIs, with high PD-L1 expression associated with worse outcomes but better response to ICIs (18–20).

Chemotherapy may enhance immunotherapy efficacy by reducing the immunosuppressive effects of the tumor microenvironment, facilitating cross-presentation of tumor antigens, and promoting immune cell infiltration into the tumor core (21–23). The combination of ICIs with chemotherapy has shown efficacy in the treatment of ICC. **Table 1** summarizes clinical trials that demonstrated significantly prolong overall survival (OS) with the combination of ICIs and chemotherapy (18, 24–30). The TOPAZ-1 phase III clinical trial (ClinicalTrials.gov identifier NCT03875235) indicated gemcitabine/cisplatin plus durvalumab as the preferred first-line treatment for advanced BTC (26). The results showed significantly increased estimated 24-month OS rates in the gemcitabine/cisplatin plus PD-L1 inhibitor durvalumab treatment group compared with the placebo group (24.9% vs. 10.4%). The KEYNOTE-966 global phase III clinical trial, investigating the efficacy of pembrolizumab in combination with gemcitabine and cisplatin for previously untreated metastatic or unresectable BTC patients (30), found significantly longer median OS for the pembrolizumab group than for the placebo group (12.7 vs. 10.9 months). Pembrolizumab plus gemcitabine and cisplatin is therefore a promising treatment option for patients with unresectable BTC.

**Table 1:** Outcomes of recent clinical trials of immune check point inhibitors in combination with chemotherapy.

Author	Trial number	Phase	Treatment Arm(s)	Patients	OS(Months)
Ueno et al. (18)	JapicCTI-153098	I	Nivolumab vs Nivolumab + GC	30 vs 30	5.2 vs 15.4
Feng et al. (24)	NCT03311789	II	Nivolumab + GC	27	8.5
Sahai et al. (25)	NCT03101566	II	Durvalumab + GC vs Nivolumab + Ipilimumab	35 vs 33	10.6 vs 8.2
Oh et al. (26)	NCT03046862	II	Durvalumab + tremelimumab + GC vs Durvalumab + GC	47 vs 49	20.2 vs 18.7
Oh et al. (26)	TOPAZ-1 NCT03875235	III	Durvalumab + GC vs Placebo + GC	341 vs 344	12.8 vs 11.5
Chen et al. (27)	NCT03486678	II	Camrelizumab + GEMOX	38	11.8
Chen et al. (28)	NCT03092895	II	Camrelizumab + GEMOX or FOLFOX	92	12.4
Li et al. (29)	NCT03796429	II	Toripalimab+ gemcitabine+S1	50	15
Kelley et al. (30)	NCT04003636	III	Pembrolizumab + GC vs GC	533 vs 536	12.7 vs 10.9

GC, gemcitabine and cisplatin chemotherapy; OS, overall survival; GEMOX, gemcitabine and oxaliplatin chemotherapy; FOLFOX, 5-fluorouracil, leucovorin and oxaliplatin chemotherapy.

Furthermore, there is a strong biological rationale for combining ICIs with tyrosine kinase inhibitors (TKIs) (31, 32). Zhu et al. investigated the efficacy of PD-1 inhibitors combined with TKI lenvatinib and GEMOX chemotherapy as first-line treatment in advanced ICC, and reported a median OS and progression-free survival of 14.3 (95% CI: 11.3–not reached) and 8.63 (95% CI: 7.17–11.6) months, respectively (33). These results suggest that the combination of ICIs with chemotherapy and TKIs is another promising approach for the treatment of ICC, which will be investigated in future clinical studies.

The rapid development of new therapies, including local therapy, targeted therapy, and immunotherapy, has brought new hope for the treatment of advanced ICC. For patients with advanced ICC, conversion of an unresectable tumor is critical for improving outcomes. Accurate molecular typing, sensitivity to targeted therapy, and personalized treatment are therefore indispensable in the management of ICC. In this case, NCCN guidelines were used to develop an individualized treatment plan with a multimodality approach, including combined immunotherapy and chemotherapy, which allowed successful tumor conversion, and subsequent tumor resection. The patient gained meaningful survival outcomes. This case report suggests that for patients with advanced ICC who respond to a combination of chemotherapy and immunotherapy, radical surgery may become possible, which corresponds with other studies that have demonstrated encouraging anticancer activity and tolerable safety profiles of the camrelizumab plus GEMOX regimen as a first-line treatment in patients with advanced ICC (27, 34).

## Conclusion

In conclusion, further surgery is feasible for patients with advanced ICC who have responded to the camrelizumab plus GEMOX regimen. However, large-scale clinical trials are still required to validate these findings.

## Data availability statement

The original contributions presented in the study are included in the article/supplementary material. Further inquiries can be directed to the corresponding author.

## Ethics statement

Written informed consent was obtained from the individual(s) for the publication of any potentially identifiable images or data included in this article. Written informed consent was obtained from the participant/patient(s) for the publication of this case report.

## Author contributions

ZZ: guarantees the integrity of the entire case and edited the manuscript. XW and HL: performed the literature research, data analysis, and text proofreading. HS: provided pathological guidance and data support. JC and HL: critically revised the manuscript. All authors contributed to the article and approved the submitted version.
